# Sex-Specific Acute Cerebrovascular Responses to Photothrombotic Stroke in Mice

**DOI:** 10.1523/ENEURO.0400-22.2023

**Published:** 2024-01-10

**Authors:** Joanna Raman-Nair, Gregory Cron, Kathleen MacLeod, Baptiste Lacoste

**Affiliations:** ^1^Neuroscience Program, Ottawa Hospital Research Institute, Ottawa, Ontario K1H 8L6, Canada; ^2^Department of Cellular and Molecular Medicine, Faculty of Medicine, University of Ottawa, Ottawa, Ontario K1H 8M5, Canada; ^3^Neurology Department, Stanford University, Stanford 94305, California; ^4^Pharmaceutical Sciences, University of British Colombia, Vancouver V6T 1Z3, British Columbia, Canada; ^5^Brain and Mind Research Institute, University of Ottawa, Ottawa, Ontario K1H 8M5, Canada

**Keywords:** cerebral blood flow, ischemic stroke, mouse, rho-kinase, sex hormones

## Abstract

Mechanisms underlying cerebrovascular stroke outcomes are poorly understood, and the effects of biological sex on cerebrovascular regulation post-stroke have yet to be fully comprehended. Here, we explore the overlapping roles of gonadal sex hormones and rho-kinase (ROCK), two important modulators of cerebrovascular tone, on the acute cerebrovascular response to photothrombotic (PT) focal ischemia in mice. Male mice were gonadectomized and female mice were ovariectomized to remove gonadal hormones, whereas control (“intact”) animals received a sham surgery prior to stroke induction. Intact wild-type (WT) males showed a delayed drop in cerebral blood flow (CBF) compared with intact WT females, whereby maximal CBF drop was observed 48 h following stroke. Gonadectomy in males did not alter this response. However, ovariectomy in WT females produced a “male-like” phenotype. Intact *Rock2^+/−^* males also showed the same phenotypic response, which was not altered by gonadectomy. Alternatively, intact *Rock2^+/−^* females showed a significant difference in CBF values compared with intact WT females, displaying higher CBF values immediately post-stroke and showing a maximal CBF drop 48 h post-stroke. This pattern was not altered by ovariectomy. Altogether, these data illustrate sex differences in acute CBF responses to PT stroke, which seem to involve gonadal female sex hormones and ROCK2. Overall, this study provides a framework for exploring sex differences in acute CBF responses to focal ischemic stroke in mice.

## Significance Statement

Very few studies have investigated disparities between sexes in post-stroke outcomes. Female-associated sex hormones and rho-kinase (ROCK) have converging roles in the regulation of cerebral blood flow (CBF), which is thought to involve endothelial nitric oxide. Moreover, the modulation of CBF by ROCK has only been explored in male mice following large ischemic strokes. Understanding sex differences in cerebrovascular pathophysiology and identifying potential mediators in CBF modulation following brain injury is vital for designing novel therapeutic strategies to promote recovery in both women and men.

## Introduction

With high energy consumption and minimal energy storage, the brain is reliant on continuous cerebral blood flow (CBF) that delivers oxygen and nutrients to maintain proper function. By limiting tissue perfusion, stroke affects brain homeostasis and vascular function and compromises neuronal health (Moskowitz et al., 2010; Iadecola and Anrather, 2011; Tymianski, 2011). Of the two major stroke classifications, ischemic and hemorrhagic, ischemic stroke accounts for ∼85% of all strokes (Heart and Stroke Foundation of Canada, 2019). Ischemic stroke results from the narrowing or occlusion of a cerebral blood vessel, leading to immediate local CBF restriction to a brain area. This is exceptionally detrimental to brain functionality as, unlike other organs, the brain has a limited capacity to store energy (Willie et al., 2014). If CBF is not restored rapidly, substantial cell death occurs in the core of the injury which cannot be salvaged, resulting in debilitating functional consequences to the patient (Heiss, 2012; Hossmann, 2012).

Biological sex markedly influences the prevalence and progression of cardiovascular diseases, including stroke (Orshal and Khalil, 2004; Krause et al., 2006; Cosgrove et al., 2007; Cowan et al., 2017). Epidemiological studies show that stroke incidence dramatically increases in women following menopause (Appelros et al., 2009; Barker-Collo et al., 2015; Wang et al., 2019; Bonkhoff et al., 2021). Post-menopausal women have higher rates of stroke compared with age-matched men, resulting in increased mortality, worse psychological outcomes, and higher rates of disability (Persky et al., 2010; Turtzo and McCullough, 2010; Ahnstedt et al., 2016; Heart and Stroke Foundation of Canada, 2019; Madsen et al., 2019). Following menopause, estrogen production by the ovaries decreases drastically, which has led to the presumption that estrogens are protective against cardiovascular disease. This is thought to be mediated by the upregulation of endothelial nitric oxide synthase (eNOS) via estrogen signaling (Simoncini et al., 2000; Miyazaki-Akita et al., 2007; Novella et al., 2012; Boese et al., 2017). Increased transcription and activation of eNOS by estrogens ultimately results in increased bioavailability of nitric oxide (NO), which induces vascular smooth muscle cell (vSMC) relaxation and thereby vasodilation. For this reason, estrogen is said to increase CBF, reduce systemic blood pressure, and protect against vascular disease (Turtzo and McCullough, 2008).

In preclinical studies, removal of gonadal female hormones through ovariectomy (Ovx) has been shown to decrease CBF following experimental ischemic stroke in rodents compared to females with their ovaries left intact; however, supplementation with estrogen to restore CBF has shown conflicting results (Yang et al., 2000; McCullough et al., 2001). Only one study has shown that CBF values of female rats are higher following experimental stroke when compared with both males and Ovx females (Alkayed et al., 1998), and the mechanisms behind this difference remain elusive. Female rats also display faster vascular remodeling of occluded vessels compared with males following a focal ischemic stroke (Yang et al., 2019). But, overall, specific knowledge on sex differences in cerebrovascular disease is limited, and the regulation of endothelial function by sex hormones in disease states is poorly understood.

Another important regulator of endothelial function and vascular tone is rho-associated coiled-coil containing protein kinase (ROCK), which is activated by the upstream effector RhoA. RhoA/ROCK signaling serves many roles, including regulation of cell contractility through inhibitory action on myosin light chain phosphatase (MLCP; Çiçek and Ayaz, 2015; Hartmann et al., 2015). ROCK directly inhibits MLCP by phosphorylating the myosin-binding subunit (MYPT), thereby inhibiting the dephosphorylation of myosin light chains and resulting in sustained contraction and calcium sensitization of vSMCs (Nunes and Webb, 2021). While both isoforms of ROCK (ROCK1 and ROCK2) are found in the brain, each has differing subcellular locations and functional roles (Julian and Olson, 2014; Çiçek and Ayaz, 2015; Hartmann et al., 2015). ROCK2 is the isoform primarily expressed in the brain and its vasculature (Pelosi et al., 2007; Julian and Olson, 2014; Niego et al., 2017). Furthermore, ROCK2, but not ROCK1, bound directly to MLCP in vSMCs (Wang et al., 2009), suggesting that ROCK2 is largely responsible for regulating vSMC contractility. ROCK2 has therefore been implicated in the pathogenesis of various vascular diseases, including hypertension (Rankinen et al., 2008; Abd-Elrahman et al., 2015; Hartmann et al., 2015), age-related vascular dysfunction (Nunes and Webb, 2021), hypoxia-induced pulmonary hypertension (Shimizu et al., 2013), as well as vascular dysfunction associated with diabetes (Soliman et al., 2012; Çiçek and Ayaz, 2015) and stroke (Lee et al., 2014; Hiroi et al., 2018). Moreover, in both peripheral (Lamping and Faraci, 2001; Nuno et al., 2007, 2009; Ahnstedt et al., 2013) and cerebral (Chrissobolis et al., 2004; Faraci et al., 2006) vessels, ROCK signaling has been implicated in sex differences in cerebrovascular reactivity. ROCK also influences vascular tone by directly inhibiting both the activation (Ming et al., 2002; Sugimoto et al., 2007) and expression (Laufs and Liao, 1998) of eNOS. Furthermore, RhoA/ROCK signaling is upregulated in human endothelial cells during hypoxia, mediating the downregulation of eNOS expression and activation (Takemoto et al., 2002; Wolfrum et al., 2004; Jin et al., 2006). ROCK activity is upregulated following ischemic stroke, contributing to increased vascular permeability and enhancing oxidative stress (Allen et al., 2010; Satoh et al., 2010; Cui et al., 2013), and inhibition of ROCK improved CBF following stroke in an endothelium-dependent manner (Shin et al., 2007; Yagita et al., 2007). Specifically pertaining to ROCK2, the selective inhibition of ROCK2 after experimental ischemic stroke dose-dependently reduced infarct volumes and limited perfusion loss in male mice (Lee et al., 2014). Male *Rock2^+/−^* mice, who exhibit constitutively enhanced eNOS expression in brain endothelial cells, also have reduced infarct volumes following middle cerebral artery occlusion (MCAo; Hiroi et al., 2018). These neuroprotective effects correlated with higher levels of NO, and furthermore, neuroprotective effects were abolished in eNOS-deficient (*eNOS^−/−^*) mice (Hiroi et al., 2018). Additionally, neuroprotective effects persisted when ROCK2 was selectively ablated from endothelial cells (Hiroi et al., 2018). Thus, ROCK signaling during ischemia, particularly involving ROCK2, may contribute to vasoconstriction and reduced CBF in the hypoxic region.

Despite this context, the mechanisms underlying cerebrovascular outcomes of focal ischemic stroke in mice are poorly understood, and the effects of gonadal sex hormones on cerebrovascular regulation in the ischemic brain have yet to be fully comprehended. Considering the overlapping roles of hormonal and ROCK-mediated regulation of vascular function, this study aims to test the hypothesis that ROCK2 is involved in acute CBF outcomes following a focal ischemic stroke, in a sex-specific manner.

## Materials and Methods

### Subjects

Male and female *Rock2^+/−^* mice and wild-type (WT) littermates were bred in-house and housed a maximum of five per cage with *ad libitum* access to food and water. Complete knock-out of ROCK2 is embryonically lethal due to thrombosis and placental dysfunction and causes impaired fetal development in those that do survive (Thumkeo et al., 2003). Therefore, only heterozygous *Rock2^+/−^* mice were bred for this study. *Rock2^+/−^* breeders were obtained as a generous gift from Dr. Zhengping Jia (University of Toronto, Canada) who generated the mice on a CD1 background, as described previously (Zhou et al., 2009; Soliman et al., 2015). Animals were maintained on Teklad Global 18% Protein Rodent Diet (Harlan Laboratories, Teklad Diets) composed of 18.6% protein, 6.2% fat, 3.5% fiber, and 44.2% carbohydrates. Mice were aged 8–10 weeks when experimental procedures were initiated. All methods and procedures were approved by the University of Ottawa's Animal Care Committee and are in accordance with the Canadian Council on Animal Care guidelines.

### Female gonadectomy

Female mice received either a sham surgery (referred to herein as “intact females”) or a bilateral Ovx. Mice were initially anesthetized with 4% isoflurane and then maintained at 2.5% isoflurane for the duration of the surgery. The back of the mouse was shaved, skin disinfected, and slow-release buprenorphine (1.2 mg/kg, Chiron Compounding Pharmacy) and 1 ml of saline (0.9%) were both administered subcutaneously prior to performing the surgery. The mouse was then placed on a pad heated to 37°C and positioned in sternal recumbency. Using a sterile scalpel, a 1 cm incision was made down the midline of the lower back. The skin was then gently separated using a blunt probe, and the ovary was visualized near the flanks, where it is attached to adipose tissue. A small incision was made in the abdominal wall, and the adipose tissue was gently pulled out of the intraperitoneal cavity along with the ovary attached. A clamp was placed just below the ovary at the uterine horn and held in place for 5–10 s, followed by excision of the ovary. The clamp remained in place for an additional 5–10 s after excision to reduce bleeding. Once the clamp was released, the adipose tissue was returned to the peritoneal cavity, and the abdominal wall incision was sutured closed with size 6-0 sutures. This exact procedure was performed again on the opposite side to remove both ovaries. Finally, the midline incision in the back was closed with autoclips, and topical bupivacaine (bupivacaine hydrochloride as monohydrate, 2%, Chiron Compounding Pharmacy) was applied to the incision site for analgesia. Mice were placed in a 37°C chamber until they woke up and were then returned to a clean home cage. Mice were monitored 4 h following the procedure and for the following 3 d in the morning to ensure proper recovery. Sham Ovx surgery was performed exactly as above, without clamping or excision of the ovaries. Mice were allowed to recover for 10–14 d before any further experimental procedures were performed.

### Male gonadectomy

Male mice received either a sham surgery (referred to herein as “intact males”) or gonadectomy (Gdx) to remove both testes. Mice were initially anesthetized with 4% isoflurane and then maintained at 2.5% isoflurane for the duration of the surgery. The scrotum of the mouse was shaved and skin disinfected, and carprofen (20 mg/kg, Rimadyl, Zoetis Canada) and 1 ml of saline (0.9%) were both administered subcutaneously prior to performing the surgery. The mouse was then placed on a pad heated to 37°C and positioned in dorsal recumbency. A 1 cm incision was made down the midline of the scrotum, and the tunica was gently separated from the skin using a blunt probe. Gentle pressure was applied to the lower abdomen of the mouse to push out both testes. A clamp was then placed on the adipose tissue visible just above the testes to restrict blood flow. After ∼10 s, a size 4-0 suture was tied loosely around the tissue behind the clamp. The clamp was then released, the knot was checked to ensure no skin was caught, and then the knot was tied tightly around the tissue. The testes were clamped one more time in front of the knotted suture and then both testicles were excised. The clamp remained in place for another 5–10 s. After removing the clamp, the excision site was monitored for any bleeding, and then the incision site was closed with size 6-0 sutures. Topical bupivacaine (2%) was applied to the incision site for analgesia, and mice were placed in a 37°C chamber until they woke up and were then returned to a clean home cage. Mice were monitored for 4 h following the procedure and for the following 2 d in the morning to ensure proper recovery. Mice also received a second subcutaneous dose of carprofen (20 mg/kg) the morning following the procedure for analgesia. Sham Gdx surgery was performed exactly as above, without clamping, tying off, or excision of the testes. Mice were allowed to recover for 10–14 d before any further experimental procedures were performed.

### Laser Doppler flowmetry and photothrombotic stroke

CBF was measured using laser Doppler flowmetry (LDF, transonic tissue perfusion monitor) in the somatosensory cortex under ketamine (100 mg/kg, Vétoquinol N.-A.) and xylazine (10 mg/kg, Nerfasin 20, Dechra Regulatory B.V.) anesthesia (K/X) administered subcutaneously as a bolus dose. A top-up dose of ketamine (25 mg/kg) and xylazine (2.5 mg/kg) was administered subcutaneously as a maintenance dose ∼30 min following the initial dose. Animal temperature was maintained with a feedback-controlled rectal thermometer and heating pad system (Harvard Apparatus Homeothermic Monitoring System) at 37°C ± 1°C. After a sufficient anesthetic plane was reached, the mouse was placed in a stereotaxic frame and an incision was made down the midline of the skull. A high-speed microdrill was then used to thin the skull to translucency at the following coordinates relative to bregma: −2.7 anterior-posterior and +4 medial-lateral. At these coordinates, an LDF probe was placed just above the skull at a 25° angle to measure blood flow. The LDF technique uses a probe composed of a laser of a specific monochromatic wavelength and a detector. The laser is reflected off red blood cells and the backscattered light creates an interference pattern on the detector surface, which measures the Doppler shift of red blood cells as they pass and provides a relative measure of perfusion (Fredriksson et al., 2007). While LDF does not give an absolute measurement of perfusion, it has high temporal resolution to measure rapid relative changes in perfusion, which can be measured reliably over time (Tajima et al., 2014). To induce photothrombotic (PT) stroke, mice received an intraperitoneal injection of the photosensitive dye rose bengal (RB, 100 mg/kg, MilliporeSigma, catalog #R3877) dissolved in phosphate-buffered saline (PBS). RB was allowed to circulate for 5 min, during which baseline LDF measurements were taken. At the end of the 5 min period, a 532 nm laser was turned on for 10 min at a distance of 3 cm from the skull at the same coordinates used for LDF measurements. Photoactivation of the light-sensitive RB results in production of singlet oxygen leading to endothelium damage and platelet aggregation forming a thrombus (Semyachkina-Glushkovskaya et al., 2017). Post-stroke LDF measurements were then taken at the same coordinates for 30 min. Post-stroke blood flow measurements were normalized to baseline for each individual mouse for statistical analysis. At the end of the procedure, mice received a dose of buprenorphine (0.1 mg/kg, Vetergesic buprenorphine hydrochloride, Ceva Animal Health) administered subcutaneously for analgesia and 1.5 ml of subcutaneous saline (0.9%) to rehydrate the animal. Finally, topical bupivacaine (2%) was applied to the incision site for analgesia, and mice were left in a 30°C incubator for ∼4 h until the anesthesia had worn off and normal activity resumed.

### Follow-up laser Doppler flowmetry measurements

Additional 30 min LDF measurements were acquired at 48 h and 1 week post-stroke. Mice were anesthetized with ketamine (100 mg/kg) and xylazine (10 mg/kg), with no maintenance dose being required. Mice were again placed in a stereotaxic frame, and an LDF probe was placed at a 25° angle at the same coordinates used for all previous LDF measurements and PT stroke induction: −2.7 anterior-posterior and +4 medial-lateral. No additional thinning of the skull was performed. Animal temperature was maintained with a rectal thermometer and heating pad at 37°C ± 1°C. At the end of the procedure, mice received 1.5 ml of subcutaneous saline (0.9%), topical bupivacaine (2%) was applied to the incision site, and mice were left in a 30°C incubator to recover for 4 h.

### Infarct volumes

For quantification of infarct volumes, in vivo magnetic resonance imaging (MRI) was performed 48 h after PT stroke induction using a 7 T GE/Agilent MR (University of Ottawa pre-clinical imaging core facility). Mice were anesthetized for the MRI procedure using 2% isoflurane. A 2D fast spin echo (FSE) pulse sequence was used for the imaging, with the following parameters: slice thickness, 0.5 mm; spacing, 0 mm; field of view, 2.5 cm; matrix, 256 × 256; echo time, 41 ms; repetition time, 7,000 ms; echo train length, 8; bandwidth, 16 kHz; and fat saturation. Stroke lesions demonstrated hyperintensity. MRI images were loaded in the Fiji software (https://imagej.net/software/fiji/), and infarct volumes were quantified using a custom script for outlining the infarct perimeter. All infarct volumes were quantified twice to obtain an average of two measurements for each animal. Subsequent LDF measurements for this time-point were taken at least 1 h following isoflurane exposure.

### Western blot

Baseline protein levels of ROCK were assessed 48 h following PT stroke or sham surgery (no laser irradiation) in naive (no gonadectomy or sham surgery) WT male and female mice. Following cervical dislocation, brains were rapidly extracted and placed in cold PBS, and the cerebral cortex was microdissected to obtain tissue encompassing the entire infarct as well as the peri-infarct region. This cortical tissue was immediately placed on dry ice and then stored at −80°C until tissue preparation. Cortical tissue was mechanically dissociated in RIPA buffer (150 mM NaCl, 12 mM sodium deoxycholate, 3.5 mM SDS, 50 mM Tris, Triton X-100 1%v/v, pH 8.0) with protease and phosphatase inhibitors and homogenized via several repetitions of vortexing followed by ice-cold sonication. Samples were centrifuged at 19,000 × *g* for 20 min at 4°C and soluble proteins were collected in the supernatant. The protein concentration of these samples was quantified using Pierce BCA Protein assay (Thermo Scientific, catalog #23227). 30 μg of each protein sample was loaded into the wells of 12% acrylamide gels (TGX Stain-Free FastCast 12% Starter Kit, Bio-Rad, catalog #1610184) and separated in running buffer (in mM, 35 SDS, 250 Tris, 1,865 glycine) by a constant current of 80 V for 30 min through the stacking gel and then increased to 120 V for 60 min through the separating gel. After a 1 min UV activation of the gels, the proteins were then transferred to a PVDF membrane (0.2 µm, Bio-Rad, catalog #1620177) in ice-cold transfer buffer (48 mM Tris 48, 38 mM glycine, methanol 20% v/v) for 105 min at 400 mAmps. After the transfer, the stain-free membrane was imaged to quantify the total protein transferred. The membranes were then blocked with 5% w/v skim milk prepared in TBST (50  mM Tris, 150 mM NaCl, Tween 20 1% v/v) for 1 h at room temperature. The membrane was then immediately incubated with primary antibodies raised against either ROCK1 (1:1,000, Abcam, catalog #ab134181) or ROCK2 (1:5,000, Abcam, catalog #125025) prepared in 1% w/v skim milk-TBST overnight at 4°C. The membranes were then washed with TBST (4 × 10 min) and incubated at room temperature for 1 h with an HRP-conjugated secondary antibody (1:10,000, Fisher Scientific, catalog #PR-W4011), also prepared in 1% skim milk-TBST. After a final wash in TBST (4 × 10 min), the proteins were detected by enhanced chemiluminescence (Pierce ECL Western Blotting Substrate, Thermo Scientific, catalog #32109) and imaged with the Bio-Rad ChemiDoc MP Imaging System. For analysis, bands were normalized to the total tryptophan-containing protein content in the respective lane of the stain-free membrane.

### ROCK activity assay

To assess ROCK activity in the brain after stroke, we assessed homogenates from naive (no gonadectomy or sham surgery) WT male and female mice 48 h following PT stroke or sham surgery (no laser irradiation) using an ELISA-based 96-well ROCK assay kit (Cell Biolabs, catalog #STA-416). Tissue was dissected and prepared as described above for Western blotting, except the cortical tissue was homogenized in the lysis buffer specified by the kit. Protein concentration was determined using Pierce BCA Protein assay (Thermo Scientific, catalog #23227), and 100 μg of total protein was loaded into the wells, and the assay was carried out following the manufacturer's instructions. Briefly, the sample was incubated for 30 min at 30°C in the presence of recombinant MYPT1 and then immunolabeled to detect phosphorylation of MYPT1 on Thr^696^ by ROCK. The HRP-based colorimetric reaction was stopped after 10 min, and absorbance was read at 450 nm using a BioTek Gen5 microplate reader (Agilent).

### Statistical analysis

To analyze post-stroke CBF values, measurements were normalized to pre-stroke baseline values after RB was injected, but before laser illumination. To do this, the percent change in CBF was measured by taking each post-stroke data point and dividing it by the mean of the entire 4 min baseline recording and multiplying that value by 100%. Each baseline data point was also divided by the mean of the entire 4 min baseline recording and multiplied by 100%. Therefore, normalized pre-stroke baseline values are always 100%, and post-stroke values measure the change in CBF from baseline values. Statistical analyses were performed using GraphPad Prism software (version 9.2.0). Hyperacute time-points were analyzed using a repeated-measures two-way ANOVA. Due to animal loss, comparisons of 48 h and 1 week data points were analyzed using a two-way ANOVA. Relative protein levels obtained from Western blot and relative ROCK activity levels obtained from the ROCK activity assay were compared using two-way ANOVAs.

## Results

### Sex differences in CBF following PT stroke in the somatosensory cortex

**Figure 1.**
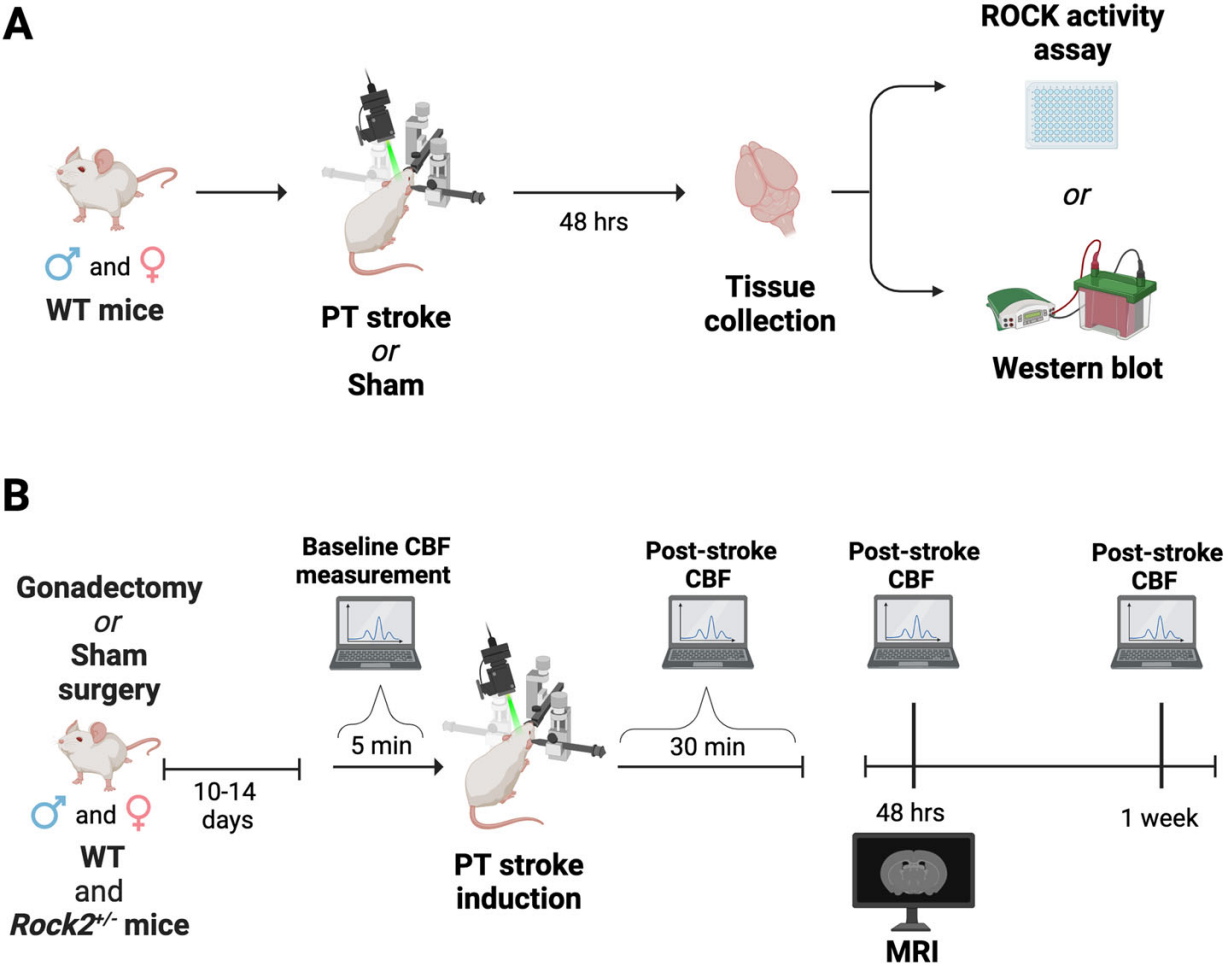
Experimental design and timeline. ***A***, Naive (not gonadectomized) adult male and female WT mice were subjected to photothrombotic (PT) stroke in the somatosensory cortex or subjected to a comparable sham surgery. 48 h following stroke, brain tissue encompassing the infarct and the peri-infarct was isolated for Western blot or ROCK activity assay analyses. ***B***, *Rock2^+/−^* male and female mice and WT littermates received a gonadectomy or comparable sham surgery and were allowed to recover for 10–14 d prior to further procedures. CBF measurements were taken using laser Doppler flowmetry (LDF) immediately before and after PT stroke induction, as well as at 48 h and 1 week post-stroke. These animals also received a magnetic resonance imaging (MRI) scan at the 48 h time-point following PT stroke. *Figure created using* *BioRender*.

**Figure 2.**
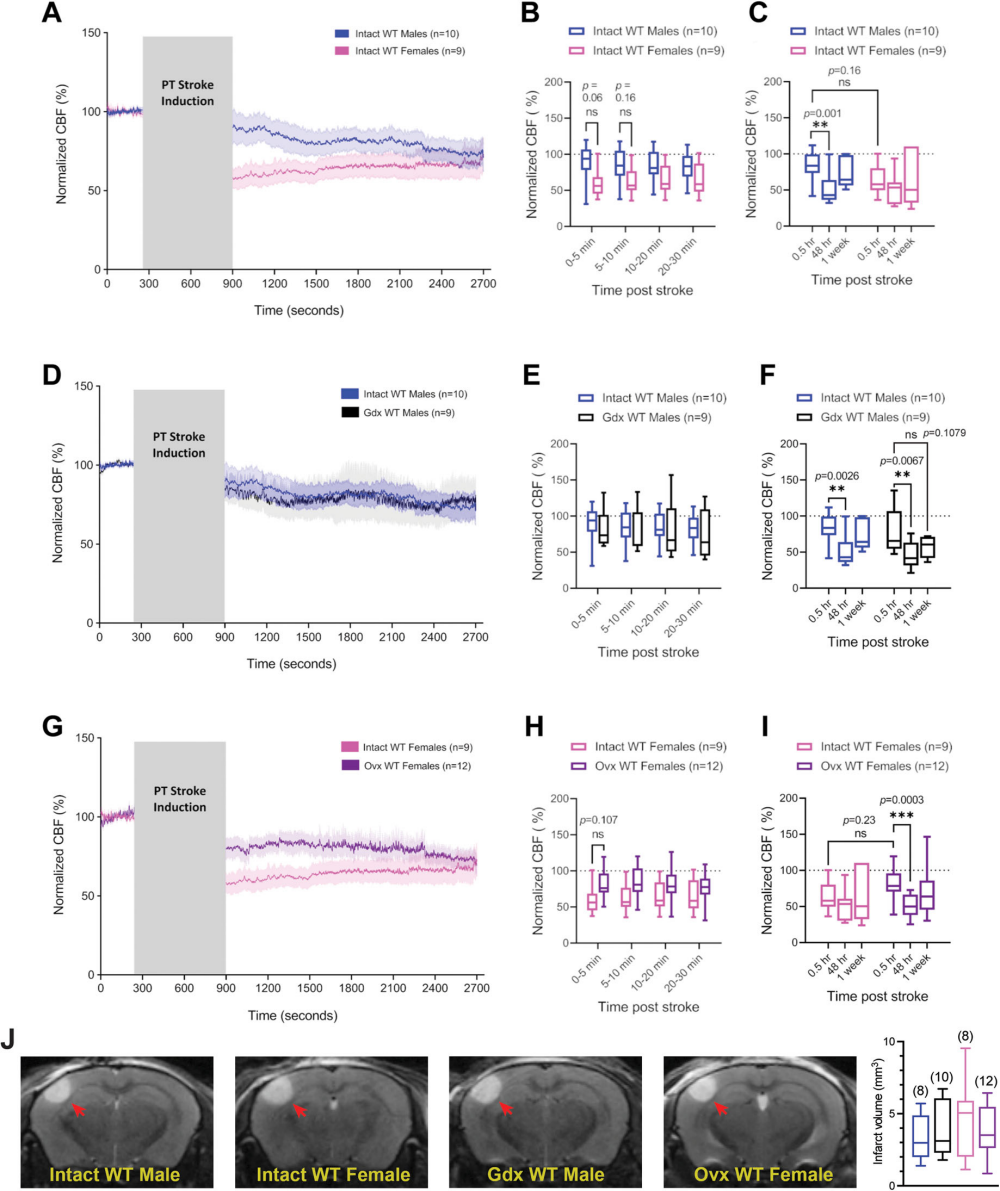
Sex differences and the influence of gonadal sex hormones in WT mice on CBF outcomes following PT stroke in the somatosensory cortex. ***A***, CBF measured by LDF under K/X anesthesia in intact WT male and female mice before and after PT stroke. Gray box indicates time passed during laser irradiation for stroke induction. ***B***, Averaged CBF measurements of hyperacute time-points during the 30 min period immediately following stroke induction. ***C***, Averaged 30 min recordings of normalized CBF measured immediately (0.5 h), 48 h, and 1 week post-stroke. ***D***, CBF measured by LDF under K/X anesthesia in intact and Gdx WT males before and after a PT stroke. Gray box indicates time passed during laser irradiation for stroke induction. ***E***, Averaged CBF measurements of hyperacute time-points during the 30 min period immediately following PT stroke induction. ***F***, Averaged 30 min recordings of normalized CBF measured immediately (0.5 h), 48 h, and 1 week post-stroke. ***G***, CBF measured by LDF under K/X anesthesia in intact and Ovx WT females before and after a PT stroke. Gray box indicates time passed during laser irradiation for stroke induction. ***H***, Averaged CBF measurements of hyperacute time-points during the 30 min period immediately following PT stroke induction. ***I***, Averaged 30 min recordings of normalized CBF measured immediately (0.5 h), 48 h, and 1 week post-stroke. All post-stroke CBF values are normalized to pre-stroke baseline values. Traces represent average normalized CBF of all animals in the respective group. Shaded areas above and below traces represent SEM. ***J***, Left, Representative MRI scans of infarcts (red arrows) imaged 48 h post-stroke. Right, Quantification of total infarct volumes measured in all experimental groups from MRI scans taken 48 h following PT stroke induction in the somatosensory cortex. All bar graphs are whisker boxes (min to max, center line indicating median). **p < 0.01, ***p < 0.001 (2-way ANOVA and Sidak's *post hoc* test).

Over the 30 min period following a PT stroke in the somatosensory cortex (Fig. 1), average CBF in intact WT males dropped to 85.13% ± 2.48 (mean ± SD) of pre-stroke baseline values, while CBF in intact WT females dropped to 62.78% ± 2.75 of baseline values (Fig. 2*A*). Although no significant difference in average CBF was measured between sex groups during the first 30 min post-stroke (Fig. 2*C*), individual curves for each group (Fig. 2*A*) showed a phenotypic difference. Indeed, intact WT females displayed an immediate reduction in CBF following stroke which stayed relatively stable throughout the 30 min recording, whereas intact WT males showed a noticeable delay in CBF drop. Because of this apparent group separation, we broke down the 30 min period into smaller sections, herein referred to as “hyperacute time-points” (Fig. 2*B*). A noticeable difference between groups, albeit not significant (*p *= 0.0635), was observed during the first 5 min following stroke, during which intact WT males had average CBF values at 88.60% ± 27.73 from baseline, while intact WT females averaged at 59.34% ± 19.47. Interestingly, when measured at 48 h following stroke, intact WT males showed a significant decrease in CBF values compared with values recorded immediately post-stroke (Fig. 2*C*). A further drop in CBF values was not observed in intact WT females, and there was no difference within or between groups at the 1 week time-point (Fig. 2*C*). MRI scans performed at the 48 h time-point following PT stroke did not reveal any difference in lesion size between these groups (Fig. 2*J*).

### Contribution of gonadal sex hormones to sex differences in CBF following PT stroke

To determine the role of gonadal sex hormones on CBF outcomes following PT stroke, we compared post-stroke CBF between gonadectomized (Gdx) or ovarectomized (Ovx) mice and intact mice that received a sham surgery. At any hyperacute time-point following stroke, no difference in CBF was found between Gdx and intact WT males (Fig. 2*D*,*E*). Furthermore, just as observed in intact WT males, Gdx WT males showed a significant decrease in CBF values at 48 h post-stroke (Fig. 2*F*). No significant difference was detected in CBF at the 1 week time-point within or between groups, while a trend to spontaneous recovery was lost in Gdx WT males (Fig. 2*F*). In females, CBF values in Ovx WT females dropped to an average of 80.22% ± 20.64 from pre-stroke baseline values during the full 30 min post-stroke period. This is higher than what was observed in intact WT females, although this difference was not statistically significant (Fig. 2*G–I*). Although there was an apparent separation between CBF traces immediately post-stroke (Fig. 2*G*), no significant difference was noted between groups at any hyperacute time-point (Fig. 2*H*). Interestingly, similar to observations made in intact and Gdx WT males, Ovx WT females showed a significant decrease in CBF values at 48 h post-stroke (Fig. 2*I*). No differences in infarct volumes were found between groups when measured 48 h after PT stroke by MRI (Fig. 2*J*).

### Sex-specific effects of ROCK2 haploinsufficiency on CBF following PT stroke

**Figure 3.**
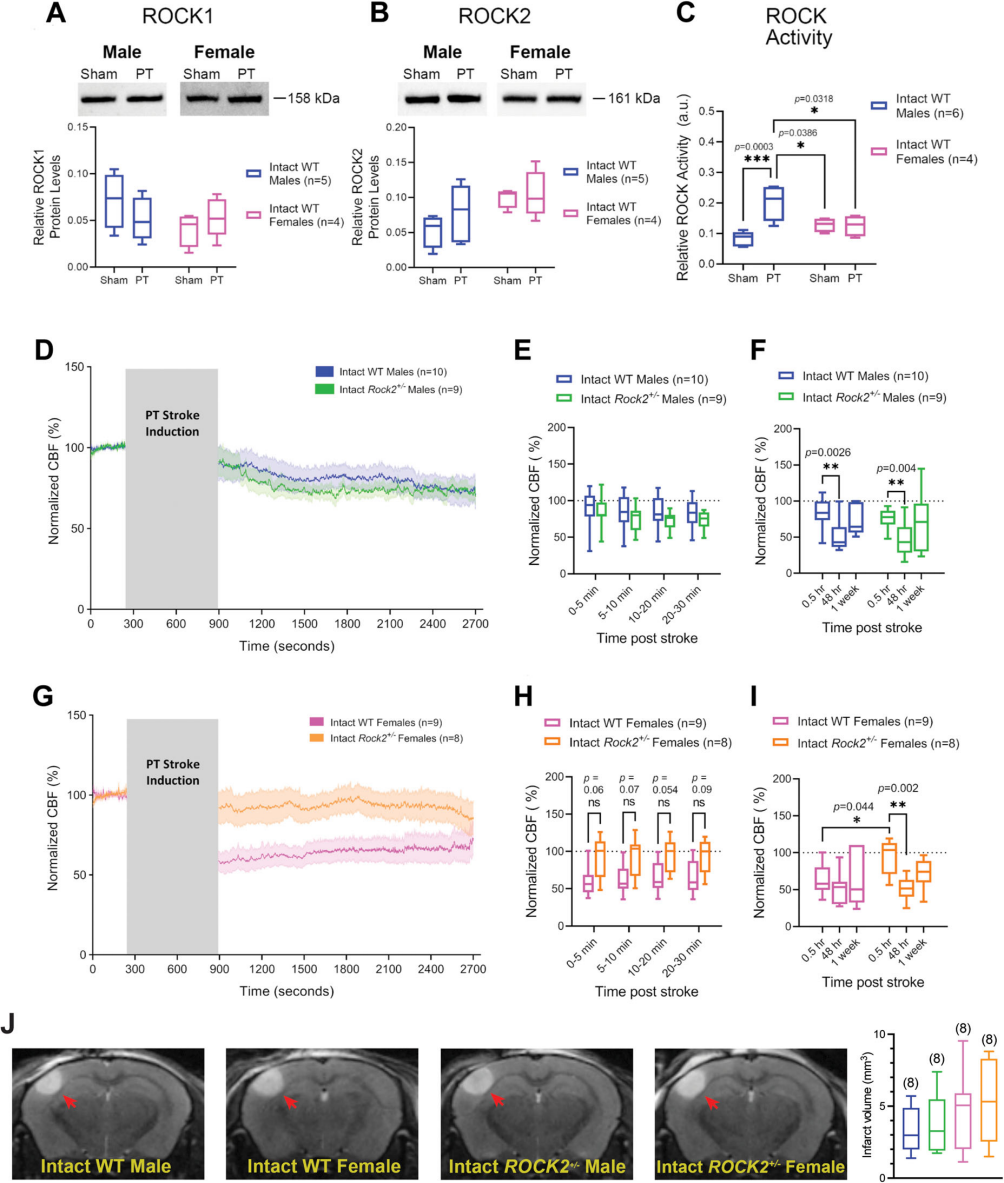
ROCK2 haploinsufficiency is associated with altered CBF outcomes in female but not male mice following PT stroke in the somatosensory cortex. ***A***, Protein levels of ROCK1 and (***B***) of ROCK2 detected by Western blot in cortical tissue from the infarct and peri-infarct region 48 h following PT stroke induction (or sham surgery) in the somatosensory cortex of naive intact WT mice. ***C***, ROCK activity assessed by ELISA measuring phosphorylation of MYPT1 at residue Thr^696^. Tissue was collected 48 h following PT stroke (or sham surgery) from naive intact WT male and female mice. ***D***, CBF measured by LDF under K/X anesthesia in intact WT and *Rock2^+/−^* male mice before and after a PT stroke. Gray box indicates time passed during laser irradiation for stroke induction. ***E***, Averaged CBF measurements of hyperacute time-points during the 30 min period immediately following PT stroke induction. ***F***, Averaged 30 min recordings of normalized CBF measured immediately (0.5 h), 48 h, and 1 week post-stroke. ***G***, CBF measured by LDF under K/X anesthesia in intact WT and *Rock2^+/−^* female mice before and after a PT stroke. Gray box indicates time passed during laser irradiation for stroke induction. ***H***, Averaged CBF measurements of hyperacute time-points during the 30 min period immediately following PT stroke induction. ***I***, Averaged 30 min recordings of normalized CBF measured immediately (0.5 h), 48 h, and 1 week post-stroke. All post-stroke CBF values are normalized to pre-stroke baseline values. Traces represent average normalized CBF of all animals in the respective group. Shaded areas above and below traces represent SEM. ***J***, Left, Representative MRI scans of infarcts (red arrows) imaged 48 h post-stroke. Right, Quantification of total infarct volumes measured in all experimental groups from MRI scans taken 48 h following PT stroke induction in the somatosensory cortex. All bar graphs are whisker boxes (min to max, center line indicating median). ***p < 0.001 **p < 0.01, *p < 0.05 (2-way ANOVA and Sidak's *post hoc* test).

ROCK protein and activity levels were assessed in peri-infarct cortical tissue from intact WT male and female mice 48 h following PT stroke induction (or sham surgery; see Fig. 1). Western blot analysis showed no difference in relative protein levels of either ROCK1 (Fig. 3*A*) or ROCK2 (Fig. 3*B*) between stroke and sham-treated animals of both sexes. However, an ELISA assay testing the phosphorylation of MYPT1, a downstream substrate of ROCK, showed that intact WT males have higher ROCK activity following PT stroke than sham-operated WT males (Fig. 3*C*). WT males with a PT stroke also had higher ROCK activity compared with intact WT females. ROCK activity did not increase in intact females 48 h after PT stroke (Fig. 3*C*).

When comparing genotypes, intact *Rock2^+/−^* and WT male mice both displayed similar CBF values at hyperacute time-points following PT stroke (Fig. 3*D*,*E*). Furthermore, as observed in intact WT males, intact *Rock2^+/−^* males also showed a significant decrease in CBF 48 h post-stroke (Fig. 3*F*). At the 1 week time-point, no difference in CBF was found between groups (Fig. 3*F*). When comparing genotypes of female mice, intact *Rock2^+/−^* females displayed significantly higher average CBF compared with their intact WT counterparts during the 30 min period following PT stroke induction (Fig. 3*G–I*). Differences between groups did not reach statistical significance when this phase was broken down into smaller intervals (Fig. 3*H*). Intact *Rock2^+/−^* females also showed a significant decrease in CBF 48 h post-stroke compared to intact WT females (Fig. 3*I*). There was no difference in infarct volume between these groups when measured 48 h after stroke (Fig. 3*J*).

### Interactions between gonadal sex hormones and ROCK2 in CBF outcomes after PT stroke

**Figure 4.**
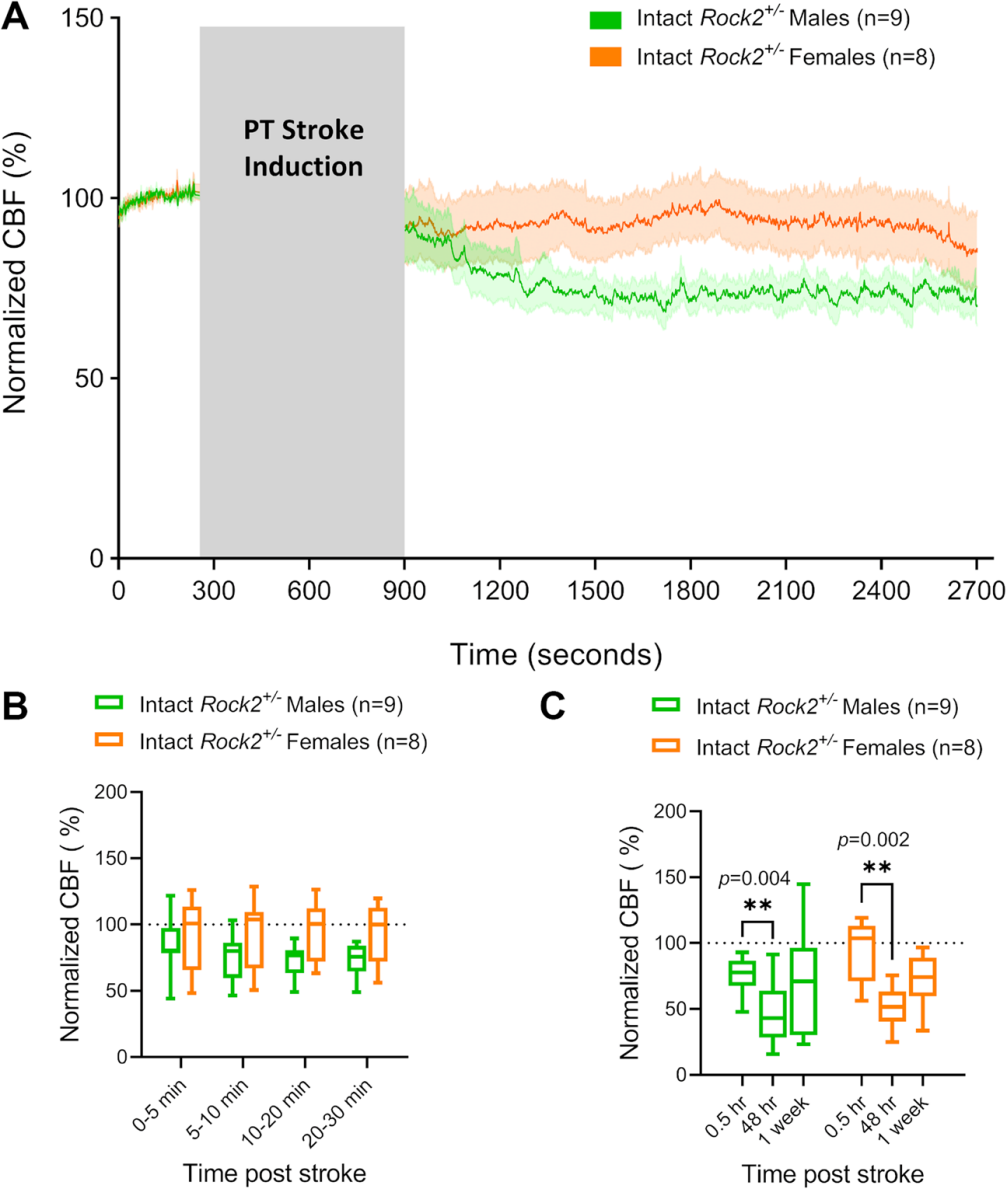
Differences between intact *Rock2^+/−^* male and female mice in CBF after PT stroke in the somatosensory cortex. ***A***, CBF measured by LDF under K/X anesthesia in intact *Rock2^+/−^* male and female mice before and after a PT stroke. Gray box indicates time passed during laser irradiation for stroke induction. Post-stroke CBF values are normalized to pre-stroke baseline values. Traces represent average normalized CBF of all animals in the respective group. Shaded areas above and below traces represent SEM. ***B***, Averaged CBF measurements of hyperacute time-points during the 30 min period immediately following PT stroke induction. ***C***, Averaged 30 min recordings of normalized CBF measured immediately (0.5 h), 48 h, and 1 week post-stroke. Bar graphs are whisker boxes (min to max, center line indicating median). **p < 0.01 (2-way ANOVA and Sidak's *post hoc* test).

**Figure 5.**
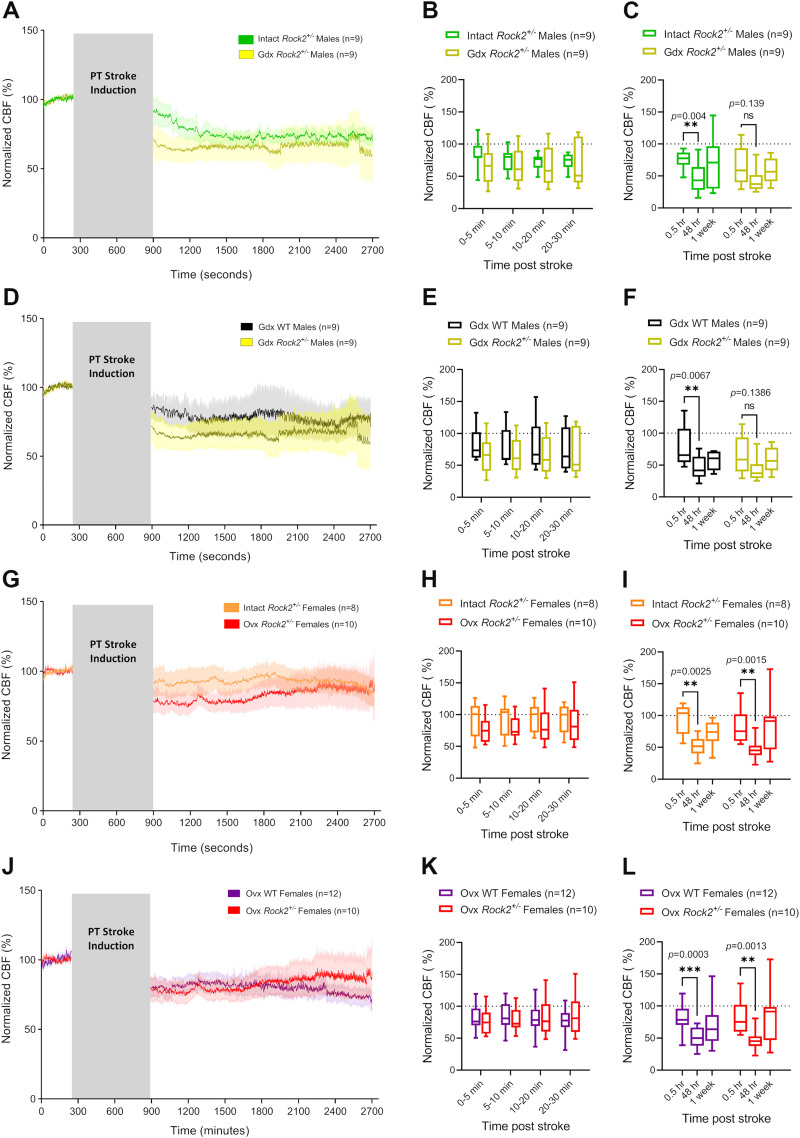
Gonadectomy does not significantly alter CBF responses to PT stroke in *Rock2^+/−^* male or female mice. ***A***, CBF measured by LDF under K/X anesthesia in intact and Gdx *Rock2^+/−^* males before and after a PT stroke. ***B***, Averaged CBF measurements of hyperacute time-points during the 30 min period immediately following PT stroke induction. ***C***, Averaged 30 min recordings of normalized CBF measured immediately (0.5 h), 48 h, and 1 week post-stroke. ***D***, CBF measured by LDF under K/X anesthesia in Gdx WT and *Rock2^+/−^* males before and after a PT stroke. ***E***, Averaged CBF measurements of hyperacute time-points during the 30 min period immediately following PT stroke induction. ***F***, Averaged 30 min recordings of normalized CBF measured immediately (0.5 h), 48 h, and 1 week post-stroke. ***G***, CBF measured by LDF under K/X anesthesia in intact and Ovx *Rock2^+/−^* females before and after a PT stroke. ***H***, Averaged CBF measurements of hyperacute time-points during the 30 min period immediately following PT stroke induction. ***I***, Averaged 30 min recordings of normalized CBF measured immediately (0.5 h), 48 h, and 1 week post-stroke. ***J***, CBF measured by LDF under K/X anesthesia in Ovx WT and *Rock2^+/−^* female mice before and after a PT stroke. ***K***, Averaged CBF measurements of hyperacute time-points during the 30 min period immediately following PT stroke induction. ***L***, Averaged 30 min recordings of normalized CBF measured immediately (0.5 h), 48 h, and 1 week post-stroke. All post-stroke CBF values in ***A***, ***D***, ***G***, and ***J*** are normalized to pre-stroke baseline values. Gray boxes indicate time passed during laser irradiation for stroke induction. Traces represent average normalized CBF of all animals in the respective group. Shaded areas above and below traces represent SEM. Bar graphs are whisker boxes (min to max, center line indicating median). ***p < 0.001, **p < 0.01 (2-way ANOVA and Sidak's *post hoc* test).

No statistical difference was detected in CBF following PT stroke between intact *Rock2^+/−^* males and intact *Rock2^+/−^* females, yet a trend toward higher CBF could be observed in females during the hyperacute phase (Fig. 4*A*,*B*). Both intact *Rock2^+/−^* males and females showed a significant drop in CBF 48 h post-stroke, with similar patterns at other time-points (Fig. 4*C*). Removal of gonadal sex hormones in *Rock2^+/−^* males did not change the CBF response to stroke in the hyperacute phase (Fig. 4*A*,*B*). Gdx *Rock2^+/−^* males also showed a decrease in CBF values at 48 h post-stroke, although this was not statistically significant due to higher variability (Fig. 5*C*). Removal of gonadal sex hormones in *Rock2^+/−^* females did not change the CBF response to PT stroke in the hyperacute phase (Fig. 5*G*,*H*). Both intact and Ovx *Rock2^+/−^* females showed significantly reduced CBF at 48 h post-stroke (Fig. 5*I*). Contrary to what was observed in WT mice, removal of gonadal hormones prior to stroke induction in *Rock2^+/−^* mice did not alter CBF outcomes in either males (Fig. 5*D–F*) or females (Fig. 5*J–L*). There were also no differences in lesion sizes between any of these groups as determined by MRI 48 h following stroke induction (data not shown).

## Discussion

This study investigates CBF outcomes following a focal (cortical) ischemic stroke in mice, with high temporal resolution. CBF was characterized by LDF immediately following stroke and up to one week in intact and gonadectomized male and female *Rock2^+/−^* mice and their WT littermates. This study provides novel insight into sex differences in acute CBF responses to brain ischemia in a preclinical model. We report marked differences between male and female mice, in which gonadal sex hormones and ROCK2 appear to play a role. Further research is required to fully elucidate the mechanisms involved in this complex pathway.

### CBF outcomes in intact wild-type male and female mice following PT stroke

Intact WT males displayed a phenotypic delay in CBF drop during the hyperacute 30 min period post-stroke when compared with intact WT females. Intact WT males also showed a further reduction in CBF at the 48 h time-point post-stroke, whereas intact WT females showed no change at this time-point. This apparent sex difference may be due to several factors, including the mechanisms of this particular stroke model. PT stroke induction involves initiation of the coagulation cascade, resulting in thrombus formation. In both animal and human studies, the female sex has shown to have higher levels of circulating platelets, and furthermore, platelets from females display higher reactivity and are more prone to aggregation and thrombus formation (Zwierzina et al., 1987; Green et al., 1992; Leng et al., 2004; Becker et al., 2006; Otahbachi et al., 2010; Miller et al., 2014; Friede et al., 2020). Because intact WT males appeared to take longer to reach a maximal CBF drop—exhibiting the lowest CBF values 48 h following stroke induction—it is possible that PT stroke induction may require more time for platelet aggregation to occur in males. The only study directly comparing CBF between male and female rodents following ischemic stroke demonstrated that 2 h after a transient intraluminal MCAo, intact female rats had higher CBF values and decreased infarct volumes compared with both intact males and Ovx females (Alkayed et al., 1998). In our study, higher CBF was not observed in females, which may be due to properties of the stroke model used. Transient MCAo is mechanistically different than PT stroke, as it involves ligation of the MCA for a determined amount of time followed by rapid reperfusion, and can involve a secondary injury (i.e., reperfusion injury). PT stroke involves only a slow reperfusion occurring via endogenous fibrinolysis of the thrombotic clot, which results in an incomplete reperfusion of the affected tissue. While intact female rodents appear to be protected in stroke models involving rapid reperfusion, this may not be the case for the PT stroke model.

### The contribution of gonadal sex hormones to CBF outcomes following PT stroke

Gonadal hormones were surgically removed from male and female mice a minimum of 10 d prior to stroke induction to assess the CBF response in comparison with intact (sham-operated) males and females. No difference in CBF was observed during the hyperacute phase post-stroke between Gdx and intact WT males. Similarly, Gdx WT males showed the same delay in reaching a maximal drop in CBF post-stroke as intact WT males. This suggests that gonadal male sex hormones may not be involved in short-term CBF regulation following a focal stroke in the mouse cerebral cortex. Interestingly, Ovx in WT females produced a similar response to that which was seen in both intact and Gdx WT males. Although not statistically significant, a clear separation was noted between CBF traces of Ovx and intact WT females within the 0–5 min hyperacute phase following stroke, during which Ovx WT females displayed slightly higher CBF values (as was seen in the aforementioned male groups). Moreover, Ovx WT females also showed a delayed decrease in CBF, reaching a maximal drop at 48 h post-stroke. These results indicate that gonadal female sex hormones may be implicated in the thrombosis response involved in the induction of a PT stroke. Previous research has shown that chronic 17β-estradiol treatment of cerebral microvessels exacerbated platelet aggregation in response to endothelial injury in both male and female mice (Rosenblum et al., 1985). Conversely, chronic testosterone or dihydrotestosterone (DHT) treatment in male mice increased platelet aggregation following endothelial injury to mesenteric arteries, but not cerebral pial vessels; and furthermore, it had no effect on aggregation in female mice (Rosenblum et al., 1987). Therefore, it is conceivable that removing gonadal estrogens from female mice results in reduced platelet aggregation during PT stroke induction.

### ROCK activity is upregulated in male but not female mice following a PT stroke

Relative protein levels of ROCK1 and ROCK2 appear unchanged after PT stroke in both sexes. While previous research has shown increased levels of ROCK2 protein and mRNA in male rats following bilateral carotid artery occlusion, these levels were not detected until 3 weeks post-stroke, with maximal levels detected at 6 weeks (Yan et al., 2015). In our study, Western blot analysis was performed at the 48 h time-point, which may be too soon for detectable changes in transcription/translation. Interestingly, despite no change in protein levels of ROCK1 and ROCK2, overall ROCK activity appeared elevated in intact WT males 48 h post-stroke. This is consistent with several studies showing increased ROCK activity following preclinical stroke models in male rodents (Rikitake et al., 2005; Yano et al., 2008; Wu et al., 2012). Conversely, there was no difference in ROCK activity between control and stroke WT females. Furthermore, ROCK activity in intact WT males was increased compared with both stroke and sham-treated intact WT females, suggesting that WT females have lower baseline levels of ROCK activity. This is the first time ROCK activity has been investigated in female mice in a preclinical stroke model. Higher baseline levels of ROCK in males is supported by research showing that male rodents have higher ROCK activity and are more responsive to ROCK inhibitors (Chrissobolis et al., 2004; Nuno et al., 2007).

### Sex-specific effects of ROCK2 haploinsufficiency on CBF responses to PT stroke

CBF outcomes in intact WT and *Rock2^+/−^* males were similar at all time-points, suggesting that ROCK2 may not be involved in acute CBF responses to PT stroke in male mice. While the consensus is that ROCK deletion or inhibition is neuroprotective against tissue damage following stroke, it is still unclear how ROCK is implicated in acute CBF responses to stroke. Previous research shows that male mice treated with the nonselective ROCK inhibitor fasudil had increased CBF values at baseline compared with control mice; however, there were no differences in regional CBF between groups following transient MCAo (Rikitake et al., 2005). Alternatively, nonselective ROCK inhibition did attenuate CBF deficits in WT mice subjected to distal MCAo, but not in mice lacking the gene for eNOS (*eNOS^−/−^*), suggesting an endothelial-dependent neuroprotective mechanism (Shin et al., 2007). On the other hand, selective inhibition of ROCK2 by KD025 failed to improve absolute CBF values of male mice subjected to distal MCAo but reduced the overall area of hypoperfusion (Lee et al., 2014). Finally, heterozygous deletion of ROCK2, but not ROCK1, in male mice improved absolute CBF following transient MCAo (Hiroi et al., 2018). While there is some evidence that ROCK inhibition and deletion improves CBF outcomes following stroke, this was not observed in our study. This may be due to the nature of the stroke model used, wherein PT stroke does not provide reperfusion injury and leads to smaller infarcts, limiting the sensitivity of measures of neuroprotection. This may also be the reason why no improvement in infarct volumes was observed in mice with *Rock2* haploinsufficiency. A failure to detect CBF improvements could also be due to the technique used to measure CBF. Indeed, LDF provides high temporal resolution but only in a small volume (∼1 mm^3^) surrounding the infarct core. It is thus possible that previously reported CBF improvements in *Rock2^+/−^* mice (Lee et al., 2014; Hiroi et al., 2018) were detected with better spatial resolutions.

*Rock2* haploinsufficiency in intact female mice was associated with interesting differences in CBF following PT stroke. The observed CBF outcomes may be due to sex differences in RhoA/ROCK signaling, which has been shown to be upregulated in platelets from females, correlating with platelet hyperreactivity (Schubert et al., 2016). Furthermore, platelets from male mice that harbor a platelet-specific ROCK2 deletion were shown to be less prone to aggregation and thrombosis and also had improved CBF following a thromboembolic model of MCAo compared with WT mice (Sladojevic et al., 2017). It is possible that increased RhoA/ROCK signaling in platelets in *Rock2^+/−^* females activates thrombus formation during PT stroke induction, resulting in faster vessel occlusion and therefore decreased CBF in these mice. There is also evidence that estrogen upregulates RhoA/ROCK signaling in female vessels (Li et al., 2014). As such, removing gonadal estrogens in females may lead to downregulation of this pathway in platelets, thereby reducing thrombus formation leading to the observed delayed drop in CBF.

## Conclusions and considerations

While ROCK inhibition and deletion have largely been regarded as beneficial in stroke outcomes via the promotion of vasodilation, this has only been studied in the male sex, primarily using the MCAo model. Studies showing that elevated ROCK activity may contribute to vascular reactivity to a greater extent in males than females suggest that inhibition of ROCK in females may not be as beneficial for long-term outcomes. Future experiments comparing behavioral and functional outcomes of ROCK inhibition and deletion in males and females following focal ischemic stroke will help assess whether their vasodilatory effects are beneficial in both sexes. Additionally, behavioral testing could provide valuable insight into how ROCK2 deletion and/or loss of gonadal hormones may influence post-stroke recovery.

CBF measurements in this study were performed under K/X anesthesia, which has limited effects on vasodilation compared with other vaporized anesthetics such as isoflurane (Rakymzhan et al., 2021). Indeed, isoflurane has neuroprotective properties in ischemic stroke, via modulation of eNOS and promoting vasodilation (Kehl et al., 2002; Krolikowski et al., 2006; Lu et al., 2017). However, there is still evidence that ketamine can cause vasodilation and alter CBF (Rakymzhan et al., 2021). Ideally, such experiments would be repeated in awake mice, which have been successfully performed with PT stroke using cranial window preparations in rats (Lu et al., 2014; Yang et al., 2019) and mice (He et al., 2020).

Lastly, while gonadectomy of male and female mice will drastically reduce the prevalence of sex hormones present in the circulation, it does not completely abolish hormonal signaling in tissues. While the gonads are a major source of sex hormone production, other sites of steroid hormone production cannot be ruled out. Steroid hormones produced by the adrenal glands, such as dehydroepiandrosterone and progesterone, are lipophilic and readily cross the blood–brain barrier where they can influence neuronal activity and vascular function, and can be further converted into estradiol and testosterone (Xu et al., 2022). Neurosteroids such as pregnenolone and allopregnanolone are also locally synthesized in the mitochondria of neurons and glial cells (Lin et al., 2022). Neurosteroids do not bind to the same receptors as steroid hormones; however, during chronic activation, they can exert transcriptional and nontranscriptional regulatory effects that can persist after gonadectomy (Reddy, 2010). Furthermore, aromatase, an enzyme responsible for catalyzing the conversion of testosterone into estradiol, is upregulated following stroke (Borowicz et al., 2011; Manwani et al., 2021), which may in turn influence local steroid profiles in the brain.

We thus demonstrate clear sex differences in acute CBF outcomes following stroke in a mouse model of PT stroke, a phenomenon in which ROCK2 and gonadal sex hormones appear to be play a role. Characterization of sex differences in other preclinical stroke models will be of the utmost importance to limit or prevent translational failure.

## Synthesis

Reviewing Editor: Christophe Bernard, INSERM & Institut de Neurosciences des Systèmes Decisions are customarily a result of the Reviewing Editor and the peer reviewers coming together and discussing their recommendations until a consensus is reached. When revisions are invited, a fact-based synthesis statement explaining their decision and outlining what is needed to prepare a revision will be listed below. The following reviewer(s) agreed to reveal their identity: NONE.

Reviewer 1

Thank you for providing the opportunity to review the manuscript entitled as: "Sex-specific acute cerebrovascular response to photothrombotic stroke in mice requires rho-kinase". The study examines the sex differences in the blood flow changes after induction of photothrombotic induced stroke (PT). They also look into the possible role of only one isoform of the enzyme Rho-associated coil-coil containing protein kinase, ROCK-2. Rock 2 is the prevalent isoform expressed in the brain and spinal cord. In this manuscript it has been studied by using haploinsufficient Rock2+/- mice. The highlight and advantage of this study is that both sexes have been tested. To explore the role of sex hormones, the authors have replicated the stroke model in gonadectomized/ovariectomized mice of both wild type and Rock2+/- mice. The most remarkable finding is that Rock2+/- female show less of a decrease in the blood flow post-stroke compared to intact WTs in short term (Fig 5C). However, the infarct size does not show difference across the genotypes, sexes and state of endogenous sex hormones. Nitric oxide has been quantified by staining in the area of the motor cortex that had undergone laser induced photothrombosis, which does not show much of a difference between the groups either.

The study is almost entirely descriptive. Although it is mentioned that one of the aims of the study is shedding light on mechanisms underlying stroke, the evidence provided is underwhelming. I would also like to bring up a few points:

Other major points:

1- Why only Rock2 has been studied? While Rock2 is the highly expressed isoform in the brain, Rock 1 is also present in both neuronal and non-neuronal brain cells. Since cerebral blood flow after stroke is affected by different cells, the possible role of Rho-kinase is incomplete without looking at both isoforms. I highly suggest exploring the expression level of both isoforms in response to PT by using Western blot or more quantitative methods. It is not enough to cite other studies since models are different and miss the information on female mice. It is also important to examine associated upstream and downstream elements of the pathway, Rho/ROCK/MLC or Rho/ROCK/LIM in WT mice of both sexes in PT induced stroke. If there are notable differences, then it makes sense to pursue the role of the pathway in detail using transgenic mice.

2- There is no mention of the origin of the of the Rock2+/- mouse colony. Have they been obtained from a collaboration or another source, or generated separately? What is the genetic background? Is it possible to use Rock2-/- in case that knock-out mice can survive to adult life? Apparently in some lines it is possible and in some it is not.

3- Adding pharmacological or molecular (dominant negative, conditional knock out, RNAi, etc.) can highly improve the mechanistic aspect of the study. Again, citing other studies are not sufficiently and addressing this the question of sex difference in this model is a very interesting and novel to be left out.

4- Adding a visual timeline that shows surgeries, treatments, PT-induced stroke and tissue harvest is always Visual timeline for surgery and treatments.

Reviewer 2

The current study investigates the cerebral blood flow changes following photothrombotic stroke using LDF starting immediately following stroke and up to one week following stroke in intact and gonadectomized male and female ROCK2+/- mice and their WT littermates. This study provides insights into the mechanisms involved in sex differences in CBF values following ischemia in a preclinical stroke model. Results suggest that there is a marked difference between males and females in the acute CBF responses to PT stroke, which appears to be mediated by endogenous female sex hormones and rho-kinase. There is a sex difference in CBF outcomes following stroke in this model of PT stroke in the somatosensory cortex, in which ROCK2 appears to be implicated. All groups except for Intact WT females show a delayed drop in CBF values, reaching a maximal drop in CBF at 48 hours following stroke induction. This is likely due to mechanistic differences in stroke induction, where both ROCK2 and endogenous female sex hormones, most likely estrogen, mediate platelet aggregation and thrombus formation resulting in delayed obstruction of the vessels in the infarct. This paper is very well written and clearly adds to the current literature of the sex differences and the transient effect of the ROCK2 in the CBF following stroke. Some clarifications are needed.

How do the authors interpret the recovery of the CBF to the baseline and observe no changes in the CBF in a week of the stroke induction? The time point they analyze the injury severity at the time when all the CBF differences are obtained (at 48 hours) and not able to see any differences may implicate that the CBF alone is not playing a role in informing the severity of the injury. This study is not testing the mice behavior as far as the motor function goes and this is also a limitation that should be mentioned in the discussion. The severity of injury obtained by IHC may not predict the functional deficits.

Authors are eliminating the gonadal hormones by gonadectomy in males and females. They are not performing adrenalectomy and not blocking the production of the neurosteroids in the brain. There is emerging data that shows astrocytes and neurons produce neurosteroids (ie neuro-estradiol produced in response to brain injury). I think instead of saying endogenous hormones, using the terminology as gonadal hormones would make more sense. Discussing this fact in the discussion is also very important.

Future directions are well explained but can be expanded. Is there a role of the long-term outcome measures? Is the vasodilation versus vasoconstriction regulated by ROCK2 detrimental or beneficial in either sex in the longterm?

## Author Response

We would like to sincerely thank both Reviewers for the time spent on carefully evaluating our manuscript and for their insightful feedback. Specific comments from Reviewer #1 motivated us to perform new experiments to provide a better justification for exploring ROCK2 haploinsufficiency. This allowed us to further our manuscript's impact by providing new data exploring sex differences in ROCKactivity following focal ischemic stroke in mice. Specific comments from Reviewer #2 helped us further refine our manuscript to clarify and elaborate on the current discussion points. In brief, major changes implemented to the revised version include:

- New data (sex differences in ROCK protein levels and activity following PT stroke).

- Improved introduction, discussion, and figures for a more comprehensive manuscript.

Here below are our point-by-point responses to Reviewers. Reviewer comments/critiques appear in black italic and our responses in blue:

Response to Reviewer # 1:

Thank you for providing the opportunity to review the manuscript entitled as: "Sex-specific acute cerebrovascular response to photothrombotic stroke in mice requires rho-kinase". The study examines the sex differences in the blood flow changes after induction of photothrombotic induced stroke (PT). They also look into the possible role of only one isoform of the enzyme Rho-associated coil-coil containing protein kinase, ROCK-2. Rock 2 is the prevalent isoform expressed in the brain and spinal cord. In this manuscript it has been studied by using haploinsufficient Rock2+/- mice. The highlight and advantage of this study is that both sexes have been tested. To explore the role of sex hormones, the authors have replicated the stroke model in gonadectomized/ovariectomized mice of both wild type and Rock2+/- mice. The most remarkable finding is that Rock2+/- female show less of a decrease in the blood flow poststroke compared to intact WTs in short term (Fig 5C). However, the infarct size does not show difference across the genotypes, sexes and state of endogenous sex hormones. Nitric oxide has been quantified by staining in the area of the motor cortex that had undergone laser induced photothrombosis, which does not show much of a difference between the groups either.

The study is almost entirely descriptive. Although it is mentioned that one of the aims of the study is shedding light on mechanisms underlying stroke, the evidence provided is underwhelming. I would also like to bring up a few points:

We would like to thank Reviewer #1 for her/his thorough expert review. We agree with Reviewer #1 that this manuscript was largely descriptive, and we have added new data to improve our rationale for focusing on ROCK.

Other major points:

1- Why only Rock2 has been studied? While Rock2 is the highly expressed isoform in the brain, Rock 1 is also present in both neuronal and non-neuronal brain cells. Since cerebral blood flow after stroke is affected by different cells, the possible role of Rho-kinase is incomplete without looking at both isoforms. I highly suggest exploring the expression level of both isoforms in response to PT by using Western blot or more quantitative methods. It is not enough to cite other studies since models are different and miss the information on female mice. It is also important to examine associated upstream and downstream elements of the pathway, Rho/ROCK/MLC or Rho/ROCK/LIM in WT mice of both sexes in PT induced stroke. If there are notable differences, then it makes sense to pursue the role of the pathway in detail using transgenic mice.

We agree that it is important to characterize ROCK regulation in different stroke models, and especially between sexes in these models. We have taken Reviewer #1's suggestion of performing additional experiments to assess both ROCK1 and ROCK2 protein levels after stroke by Western Blot. We have also taken the suggestion to explore downstream elements of the pathway by assessing ROCK activity via phosphorylation of MLCP. While in our PT stroke model both ROCK1 and ROCK2 protein levels appeared unchanged, we found notable differences in ROCK activity and thus believe that this new data brings valuable insight and improves the overall manuscript (see new figure 2).

Our focus on ROCK2 was motivated by a study (Wang et al., 2009) showing ROCK2 is the specific isoform involved in phosphorylation of MLCP, and thus largely responsible for vascular smooth muscle contractility, which is now better justified in the text.

2- There is no mention of the origin of the of the Rock2+/- mouse colony. Have they been obtained from a collaboration or another source, or generated separately? What is the genetic background? Is it possible to use Rock2-/- in case that knock-out mice can survive to adult life? Apparently in some lines it is possible and in some it is not.

We have addressed this by providing details about the background of the ROCK2+/- mouse colony and have provided the necessary information in the methods section. Our mice were a gift from another investigator and are on a CD1 background. A double knockout of ROCK2 has been found to be embryonically lethal, we decided to work with this haploinsufficient model. And while it is true that some studies have shown better survivability with a full ROCK2 knockout in certain mouse strains, we only had access to breeding pairs for the heterozygous knockout on a CD1 background.

3- Adding pharmacological or molecular (dominant negative, conditional knock out, RNAi, etc.) can highly improve the mechanistic aspect of the study. Again, citing other studies are not sufficiently and addressing this the question of sex difference in this model is a very interesting and novel to be left out. We agree that pharmacological or molecular intervention would be extremely valuable and improve the mechanistic aspect of the study. Indeed, exploring sex differences in CBF outcomes after stroke in an endothelial-specific ROCK2 knockout mouse and/or eNOS deficient mice would be very valuable. However, our attempt to acquire Rock2flx/flx and eNOSflox/flox mice have failed, and now with reduced capacity and limited funding this is not something we can address given the current timeline. But we are excited to carry out this work in the future and to provide more concrete mechanistic evidence.

4- Adding a visual timeline that shows surgeries, treatments, PT-induced stroke and tissue harvest is always Visual timeline for surgery and treatments.

We agree that adding a visual timeline drastically improves the understanding of the overall scope of the project. We have added a timeline of the experimental procedures at the end of the methods section in a new figure (see new Figure 1).

Response to Reviewer # 2:

The current study investigates the cerebral blood flow changes following photothrombotic stroke using LDF starting immediately following stroke and up to one week following stroke in intact and gonadectomized male and female ROCK2+/- mice and their WT littermates. This study provides insights into the mechanisms involved in sex differences in CBF values following ischemia in a preclinical stroke model. Results suggest that there is a marked difference between males and females in the acute CBF responses to PT stroke, which appears to be mediated by endogenous female sex hormones and rho-kinase. There is a sex difference in CBF outcomes following stroke in this model of PT stroke in the somatosensory cortex, in which ROCK2 appears to be implicated. All groups except for Intact WT females show a delayed drop in CBF values, reaching a maximal drop in CBF at 48 hours following stroke induction. This is likely due to mechanistic differences in stroke induction, where both ROCK2 and endogenous female sex hormones, most likely estrogen, mediate platelet aggregation and thrombus formation resulting in delayed obstruction of the vessels in the infarct. This paper is very well written and clearly adds to the current literature of the sex differences and the transient effect of the ROCK2 in the CBF following stroke. Some clarifications are needed.

We would like to thank Reviewer #2 for her/his detailed expert review of our manuscript and were pleased to read the comment that the "...paper is very well written and clearly adds to the current literature of the sex differences and the transient effect of the ROCK2 in the CBF following stroke." After carefully considering all concerns, we have clarified and modified points throughout the manuscript.

How do the authors interpret the recovery of the CBF to the baseline and observe no changes in the CBF in a week of the stroke induction? The time point they analyze the injury severity at the time when all the CBF differences are obtained (at 48 hours) and not able to see any differences may implicate that the CBF alone is not playing a role in informing the severity of the injury. This study is not testing the mice behavior as far as the motor function goes and this is also a limitation that should be mentioned in the discussion. The severity of injury obtained by IHC may not predict the functional deficits.

We would like to thank Reviewer #2 for bringing attention to this important point that was left unaddressed in the discussion. There is a lot of variability in the CBF values at the 1 week timepoint. We agree that histological assessment of the lesion alone leaves other functional aspects of dysfunction unaddressed and thus we have detailed the limitations of not assessing behavioral deficits in these mice in the discussion.

Authors are eliminating the gonadal hormones by gonadectomy in males and females. They are not performing adrenalectomy and not blocking the production of the neurosteroids in the brain. There is emerging data that shows astrocytes and neurons produce neurosteroids (ie neuro-estradiol produced in response to brain injury). I think instead of saying endogenous hormones, using the terminology as gonadal hormones would make more sense. Discussing this fact in the discussion is also very important.

The text has now been edited throughout to address this terminology issue and "endogenous hormones" has been replaced for the term "gonadal hormones" throughout the manuscript. The presence of other sources of hormones as well as the limitations of not removing them was also added to the discussion. We would like to thank Reviewer #2 for pointing out this important clarification and we believe distinguishing these points in the text greatly strengthens the discussion.

Future directions are well explained but can be expanded. Is there a role of the long-term outcome measures? Is the vasodilation versus vasoconstriction regulated by ROCK2 detrimental or beneficial in either sex in the longterm?

We have expanded the discussion to include more information on how ROCK2 regulation may implicate vascular function in the long-term. Vasodilatory effects of ROCK inhibition have been shown to be beneficial to the male sex following stroke in preclinical research, however, based on the results of our study we hypothesize this may not be the case. ROCK2 activation and regulation appears to be different in males and females, thus we hypothesize that the beneficial effect of its inhibition may not be apparent in the female sex.
